# Aberrant placental immune parameters in the feline immunodeficiency virus (FIV)-infected cat suggest virus-induced changes in T cell function

**DOI:** 10.1186/1743-422X-10-238

**Published:** 2013-07-19

**Authors:** Lyndon Bart Chumbley, Crystal E Boudreaux, Karen S Coats

**Affiliations:** 1 Department of Biological Sciences, Mississippi State University, Mississippi State, MS 39762, USA; 2 Virginia Tech Carilion Research Institute and School of Medicine, Roanoke, VA 24016, USA

**Keywords:** Feline immunodeficiency virus, Placenta, Regulatory T cells, Th17 cells, Cytokines

## Abstract

**Background:**

Immune activity during pregnancy must be tightly regulated to ensure successful pregnancy. This regulation includes the suppression of inflammatory activity that could target the semi-allogeneic fetus. Tregs are immunosuppressive; Th17 cells are pro-inflammatory. A precise balance in the two cell populations is critical to pregnancy maintenance, while dysregulation in this balance accompanies compromised pregnancy in humans and mice. FIV is known to target Tregs preferentially in the infected cat. Therefore, it may be hypothesized that FIV infection alters the placental Treg/Th17 cell balance resulting in aberrant immunomodulator expression by these cells and consequent pregnancy perturbation.

**Methods:**

RNA was purified from random sections of whole placental tissues collected from both uninfected and FIV-infected queens at early pregnancy, including tissues from viable and nonviable fetuses. Real time qPCR was performed to quantify expression of intranuclear markers of Tregs (FoxP3) and Th17 cells (RORγ); cytokine products of Tregs (IL-10 and TGF-β), Th17 cells (IL-2, IL-6, and IL-17a), and macrophages (IL-1β); and the FIV gag gene. Pairwise comparisons were made to evaluate coexpression patterns between the cytokines and between the cytokines and the virus.

**Results:**

Both FoxP3 and RORγ were reduced in placentas of infected animals. Neither infection status nor fetal viability affected placental expression of IL-1β. However, fetal nonviability was associated with reduced levels of all other cytokines. Infection and fetal nonviability impacted coexpression of various cytokine pairs. No obvious bias toward Treg or Th17 cells was observed.

**Conclusions:**

FIV infection coupled with fetal nonviability alters expression patterns of T cell cytokines. These data suggest that functionally altered placental T cell leukocyte populations may occur in the infected queen and possibly contribute to fetal nonviability.

## Background

New pediatric AIDS cases are on the decline globally, largely a result of antiretroviral prophylaxis reaching low and middle income countries. In 2011, the number of children acquiring HIV infection had declined by 43% since 2009. Sub-Saharan Africa, where pediatric disease has also declined, remains the most dominant site of pediatric AIDS cases, comprising 90% of new infections. [1]. However, in the absence of antiretroviral therapy, mother-to-child transmission of HIV occurs in 15-45% of pregnancies [2]. HIV infection of pregnant women may result in compromised pregnancy, including low birth weight babies, pre-term delivery, and enhanced incidence of spontaneous abortions [3-5]. Feline immunodeficiency virus (FIV) infection in cats is a small animal model system used to study lentivirus pathogenesis and mother-to-child transmission. FIV, like HIV, infects CD4+ T cells and other cells of the immune system leading to immunosuppression and ultimately feline AIDS [6]. FIV infection is known to impact pregnancy outcome negatively [7,8], possibly due to altered placental immunology resulting from FIV infection of placental tissues.

Regulatory T cells (Tregs), a CD4 + CD25+ T cell population, are a naturally immunosuppressive lymphocyte population characterized by the intranuclear expression of the transcriptional regulator FoxP3. Tregs are susceptible to infection by HIV [9], potentially leading to the beneficial outcome of suppression of inflammation or, detrimentally, persistent infection (reviewed in [10]). Likewise, feline Tregs are preferentially infected by FIV, due to increased expression of CXCR4 (the host co-receptor for FIV) on the surface of Tregs [11]. Due to the semi-allogeneic status of the fetus within an antigenically foreign mother, fetal Tregs are necessary to promote immunological tolerance to maternal antigens [12,13], while maternal Tregs promote tolerance of the fetus [14]. Successful pregnancy is associated with increased numbers of activated Tregs, while deficiency in Treg numbers and activity is associated with pregnancy failure [15].

Treg activity is closely tied to the activity of IL-17 producing T helper cells (Th17 cells), a proinflammatory CD4+ T cell population. The inappropriate activity of these cells is associated with autoimmunity, graft rejection, and unexplained recurrent miscarriage [16,17]. Th17 cells perform their proinflammatory role by the secretion of cytokines including IL-6, IL-17a/f, IL-21, IL-22, TNFα, and others [18,19]. IL-17, the pleiotropic immunomodulator for which the cell population is named, acts by promoting the expression of proinflammatory cytokines in other neighboring cells. TGF-β and IL-10, key cytokine products of Tregs, inhibit IL-17 expression in a dose-dependent manner [20]. In the absence of Treg activity, Th17 cell activity is unchecked and aberrantly elevated [18].

We are using the FIV-infected cat model to evaluate placental expression levels of relevant cytokine targets that are produced by Tregs, Th17 cells, and other placental cell populations [21-23]. IL-1β is a product of placental macrophages and is a key regulator in early differentiation and differential expression of Th17 cells [24-26]. IL-2, IL-6 and IL-17a are products of activated Th17 cells; IL-2 is produced broadly by activated T lymphocyte populations [27,28]. IL-10 and TGF-β are immunosuppressive products of Tregs [29,30].

We hypothesized that lentivirus infection in the placenta alters the normal gene expression of placental T lymphocytes resulting in aberrant leukocyte immunomodulation, placental immunopathology, and possibly compromised pregnancy. The first objective of this study was to assess the expression of pro- and anti-inflammatory cytokines relevant to Treg and Th17 cell function and the characteristic intranuclear markers of these cells (FoxP3 and RORγ, respectively) in sections of whole placental tissues obtained from viable and nonviable pregnancies from FIV-infected and control queens. The second objective was to correlate FIV gag expression in placental tissues with expression of cytokine RNA to determine whether virus levels impacted (or were impacted by) particular cytokine gene expression. We found that infection caused reduced expression of FoxP3 and RORγ, and that fetal nonviability was associated with reduced expression of cytokines and discordant cytokine coexpression patterns. A negative correlation between FIV gag and some cytokines occurred in placentas from infected animals. Collectively, these data suggest that dynamics or function of placental Treg and Th17 populations is disturbed during early pregnancy in FIV-infected cats, possibly predisposing nonviable pregnancy.

## Results

### Expression of Treg and Th17 intranuclear markers in placentas

**Figure 1:**
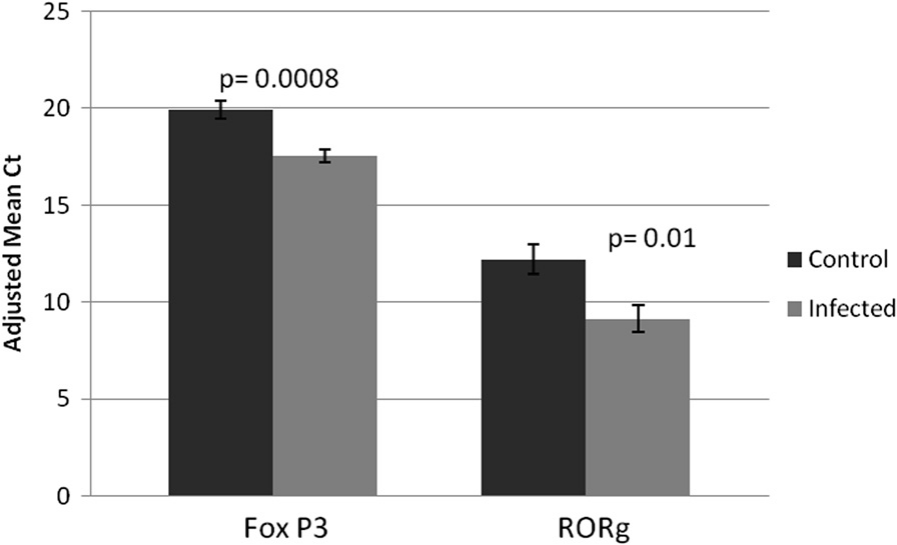
**Expression of FoxP3 and RORγ in control and infected early term placental tissue.** Real time pPCR was used to quantify the expression of FoxP3 and RORγ RNA in control (n = 10) and infected (n = 8) early term whole placental tissues. Adjusted mean Ct, represented by vertical bars, is the normalized mean Ct value subtracted from a negative endpoint (50-mean Ct). Bars are bracketed by the standard error of the mean. The data were analyzed using single factor ANOVA. P values ≤ 0.05 were considered significant.

Quantitative PCR targeting FoxP3 and RORγ, intranuclear markers for activated Tregs and Th17 cells, respectively, in a random selection of placentas from the control and infected groups (Table 1) was performed. The expression of FoxP3 (p = 0.008) and RORγ (p = 0.01) were both significantly reduced in the FIV-infected group (including placentas from three nonviable offspring), as compared to control animals (all viable fetuses) (Figure 1). When placentas from nonviable pregnancies were excluded from the analyses to allow comparison of viable pregnancies independently, significant reduction in expression of both genes was still evident in the infected group (p ≤ 0.05; data not shown).

**Table 1:** Placental samples assayed for T cell marker and cytokine gene expression

**Cat number**	**Queen infection status**	**Fetus identification**	**Placental infection status**	**Fetal viability**	**Assay**	
					**FoxP3/ ROR**	**Cytokines**
104538059	-	A	-	V	Y	
		B	-	V	Y	
		C	-	V	Y	Y
		D		V	Y	
		E	-	V		Y
104016108	-	A	-	V	Y	Y
		B	-	V	Y	Y
		C	-	V	Y	
104537284	-	A	-	V	Y	
		B	-	V	Y	Y
		C	-	V	Y	
		D	-	V		Y
104108291	-	A	-	V		Y
		B	-	V		Y
104083550	-	A	-	V		Y
		B	-	V		Y
104292779	-	A	-	V		Y
		B	-	V		Y
		E	-	NV		Y
104539276	-	A	-	V		Y
		B	-	V		Y
		F	-	NV		Y
104100373	-	A	-	V		Y
		B	-	V		Y
083556062	+	A	+	V	Y	Y
		B	+	V	Y	
		C	+	V		Y
104058035	+	A	+	V	Y	Y
		B	+	NV		Y
		C	+	NV	Y	Y
		D	+	NV		Y
104521126	+	A	+	V	Y	Y
		B	+	V	Y	
		D	+	V		Y
104005111	+	A	+	V		Y
		B	+	V		Y
		R	+	NV		Y
104280866	+	A	+	V	Y	
		B	+	NV	Y	Y
104111893	+	A	+	V		Y
		B	+	V		Y
		R	+	NV		Y

### Gene expression profiling in viable versus nonviable placental samples

**Figure 2:**
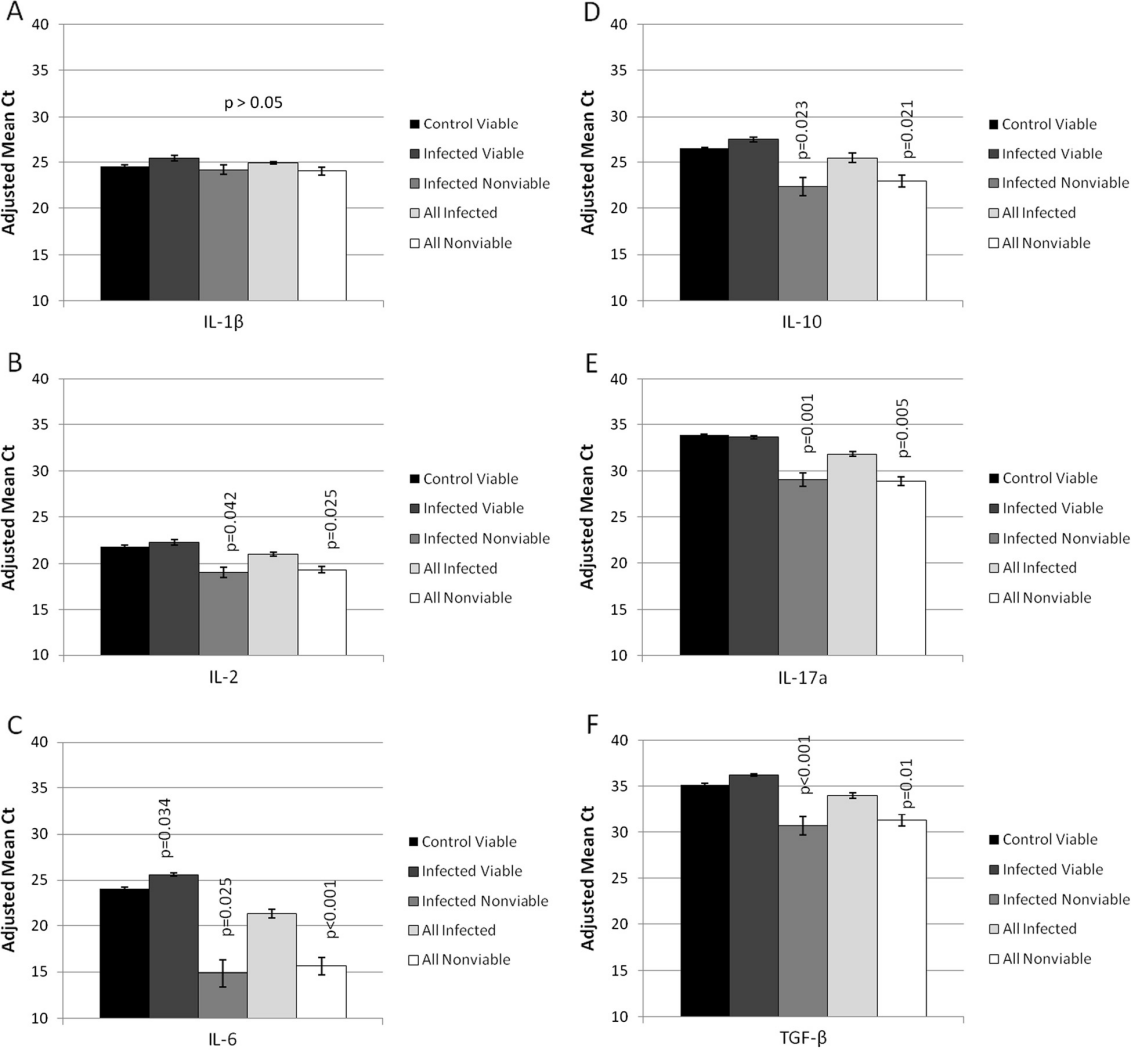
**Expression of cytokine genes in early term placental tissue from viable and nonviable offspring from control and infected queens.** Real time qPCR was used to quantify the expression of relevant cytokines in placental tissue. Cytokine gene expression was determined for samples from control queens producing viable offspring (control viable; n = 16), infected queens producing viable offspring (infected viable; n = 9), infected queens producing nonviable offspring (infected nonviable; n = 6), all infected samples combined (all infected; n = 15), and all samples from nonviable pregnancies combined (all nonviable; n = 8). Adjusted mean Ct, represented by vertical bars, is the normalized mean Ct value subtracted from a negative endpoint (60-mean Ct). Bars are bracketed by the standard error of the mean. The data were analyzed using single factor ANOVA and Wilcoxon signed-rank test. The mean Ct value for each separate group was compared to the mean Ct value for the control viable group. P values ≤ 0.05 were considered significant. **(A)** IL-1β; **(B)** IL-2; **(C)** IL-6; **(D)** IL-10; **(E)** IL-17a; **(F)** TGF-β.

Figure 2 displays gene expression profiles of pro- and anti-inflammatory cytokines for five placental groups: control queens producing viable offspring (control viable), infected queens producing viable offspring (infected viable), infected queens producing nonviable offspring (infected nonviable), all infected samples combined (all infected), and all samples from nonviable pregnancies combined (all nonviable). For statistical analyses, all groups were compared to the control viable group. There were no significant differences in IL-1β gene expression between the groups (Figure 2A). A comparison of placental tissues from control viable pregnancies to infected viable pregnancies revealed that only the expression of IL-6 (Figure 2C), a pro-inflammatory cytokine crucial for the differentiation of Th17 cells, was altered, increasing significantly (p = 0.034). (Placenta 104083550 A was not included in this analysis because it was an outlier). No other significant differences in cytokine gene expression between these two groups were detected. Treg cytokine gene expression patterns, IL-10 (Figure 2D) and TGF-β (Figure 2F), and Th17 cytokine gene expression patterns, IL-6 (Figure 2C), IL-2 (Figure 2B), and IL-17a (Figure 2E), were significantly reduced when the infected nonviable group and the all nonviable group were compared to the control viable group. Comparisons of cytokine expression in all control specimens (including those from both viable and nonviable fetuses) and all infected specimens did not differ significantly (P > 0.05; data not shown). β-actin expression did not differ significantly between any group (data not shown).

### Correlation of Cytokine Expression in Whole Placental Tissue

**Figure 3:**
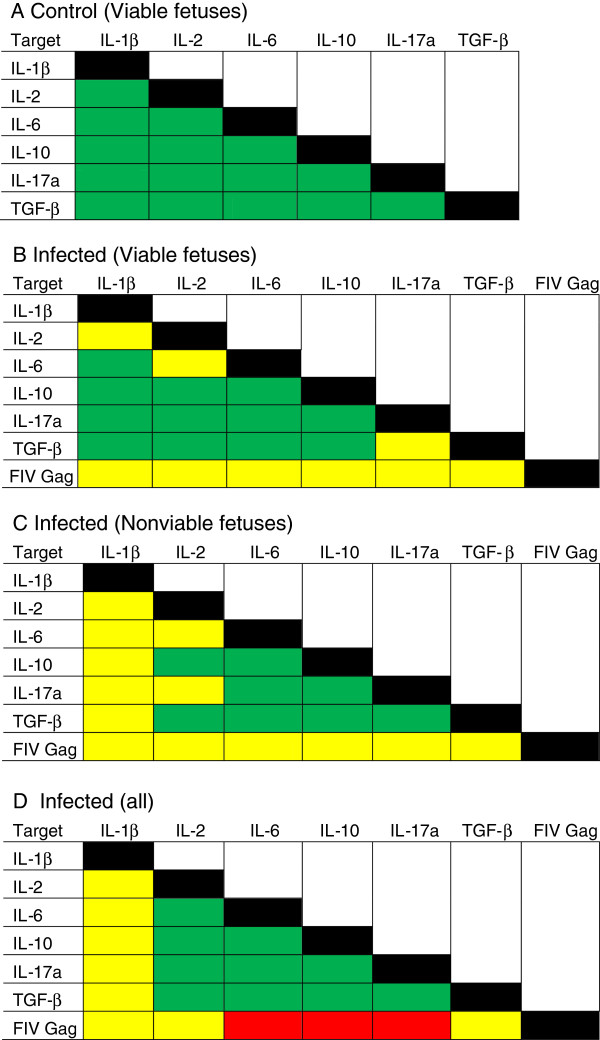
**Summary of pairwise comparisons of cytokine gene expression and FIV gag expression in early term placental tissue from viable and nonviable offspring from control and infected queens.** The expression of each gene was compared to that of all other genes. **(A)** control queens producing viable offspring (control viable; n = 16); **(B)** infected queens producing viable offspring (infected viable; n = 9); **(C)** infected queens producing nonviable offspring (infected nonviable; n = 6); **(D)** all infected samples combined (all infected; n = 15). Pairwise comparisons were analyzed using Spearman rank correlation regression analysis. P values ≤ 0.05 were considered significant. Green = positive correlation; yellow = no correlation; and red = negative correlation.

Correlation analyses were used to observe the pairwise relationships of cytokine expression within each cat. All cytokine expression data, along with FIV gag expression levels [21], were compared and the data are summarized (Figure 3). All pairwise comparisons of cytokine expression levels were positively correlated in the placentas of control queens producing viable offspring (Figure 3A). Placentas from viable infected fetuses (Figure 3B) displayed discordant (neither positively nor negatively correlated) coexpression of IL-2 versus IL-1β, IL-2 versus IL-6, and TGF-β versus IL-17a. In the nonviable infected group (Figure 3C) and the all infected group (Figure 3D), IL-1β and coexpression of all other cytokines was discordant. IL-6 versus IL-2 coexpression was discordant in the viable infected and the nonviable infected; IL17a versus IL-2 coexpression was also discordant in the nonviable infected group (Figure 3D) but positively correlated in all other groups.

FIV gag expression neither positively nor negatively correlated with expression of any cytokine in the infected viable (n = 7; Figure 3B) and infected nonviable groups (n = 6; Figure 3C). A trend toward negative correlation with some cytokines that did not reach the level of statistical significance (p > 0.05) was evident in the latter group (data not shown). When all infected placentas were combined, FIV gag was negatively correlated with the expression of IL-6, IL-10, and IL-17a (Figure 3D).

## Discussion

The Treg/Th17 paradigm of pregnancy is rapidly emerging. Numerous reports indicate that successful pregnancy is accompanied by Treg dominance in the maternal endometrium, especially early in pregnancy, while unexplained recurrent miscarriage is associated with elevated Th17 cell activity [15,17,20]. Using the FIV-infected cat to model mother-to-child transmission in utero, we reported that infection results in significantly higher rates of pregnancy failure than in uninfected animals [7,8]. In addition, infection corresponds to reduced placental expression of FoxP3 at early pregnancy, indicating that infection may diminish the number or function of Tregs in these tissues [21,23]. The preferential replication of FIV in peripheral Tregs of cats [31,32] suggests the vulnerability of this cell population to FIV infection in placental tissues as well. There are no reports of FIV infection of Th17 cells or the fate of those cells in infected animals. However, in both HIV-infected patients [33] and SIV-infected macaques [34], diminished Th17 cells accompany disease progression. In the current study, reduced placental expression of both FoxP3 and RORγ in infected queens at early pregnancy suggests Treg and Th17 suppression. In a previous report, a slight decrease in RORγ expression in placentas from FIV-infected queens at early pregnancy did not reach statistical significance [23]. This discrepancy may be attributed to different methods for quantifying the two markers; in the former study, FoxP3 and RORγ protein was quantified by measuring fluorescence intensity following immunofluorescence assays, rather than quantifying RNA. Moreover, while there was overlap in the placentas examined between the previous and current studies, there were differences in the tissues sampled. Inter-cat variability is a shortcoming of the feline model system, which has used outbred animals in study populations. The variability can mask subtle differences that may be of biological importance; such variability may have contributed to inconsistency between the two studies.

We also examined expression levels of some of the hallmark and less significant cytokines produced by Tregs (TGF-β and IL-10), Th17 cells (IL-6 and IL-17a), activated T helper cells and Th17 cells (IL-2) and macrophages (IL-1β), acknowledging that some of these cytokines are expressed by more than one of these cell types and other cell populations within the placenta, i.e. trophoblasts, NK cells, dendritic cells, B lymphocytes, and others. With the exception of IL-1β, all cytokines evaluated had decreased expression when placentas from nonviable fetuses (infected nonviable and all nonviable) were compared to placentas from uninfected queens producing viable offspring. It could be surmised that decreased placental physiology associated with fetal demise, rather than altered immune parameters, may have caused this result. However, the stability of expression of IL-1β and the housekeeping gene β-actin renders this explanation unlikely. The affected cytokines are all T cell products. Therefore, coupled with reduced expression of the two intranuclear markers, it seems probable that diminished T cell activity accompanies pregnancy failure in the cat model.

In the placenta, the cytokines evaluated are not limited to immunomodulatory function of leukocytes. Several of them are products of syncytio- and cytotrophoblasts [35] or endometrial tissues [36] and play roles in placental maturation. Cytokines such as IL-6, IL-1β, and TGF-β promote invasion and differentiation of trophoblasts, angiogenesis of trophoblastic villi, tissue remodeling during implantation, and cell migration (reviewed in [37]) [36,38]. Therefore, changes in expression of IL-6 and TGF-β could reflect altered trophoblast expression. We previously reported significantly decreased IL-6 expression in early term trophoblasts microdissected from placentas of nonviable pregnancies, including FIV-infected and control cats [39]. These data suggest that the trophoblast could be the dominant source of IL-6 in the placenta. In the same study, IL-1β expression in trophoblasts did not differ regardless of infection status or fetal viability, consistent with the present report. We have not examined the function or dynamics of macrophages in the feline placenta; therefore, the contribution of these cells to IL-1β levels in these feline tissues is unknown. Macrophages may be infected with FIV, but replication (measured by p24 concentration in tissue culture supernatant fluid) is minimal and much less efficient than in lymphocytes [40]. Based on this information, placental macrophage function and expression of macrophage products, such as IL-1β, may be insignificantly affected by the presence of the virus.

Pairwise comparisons of cytokine expression within each individual sample were done to determine how infection status and fetal viability were associated with coexpression. This method of comparison minimized concerns about inter-cat variability by allowing us to determine relationships from each specimen independently, rather than by comparing population means. In control animals, all pairwise comparisons, including those between pro- and anti-inflammatory cytokines, were positively correlated, a finding that may reflect a balance in the two cell populations with neither dominant. This finding was somewhat unexpected because it was assumed that expression of Treg and Th17 products would be inversely proportional due to the reported reciprocal relationship between these cell populations (with Treg dominance) in normal pregnancies (reviewed in [41]) and the long history of the Th1/Th2 paradigm that associated successful pregnancy with an anti-inflammatory placental microenvironment [42-45]. On the other hand, the discordant coexpression (neither a positive nor a negative correlation) of IL-2 and IL-6 in infected groups suggests the potential for imbalance in the Treg and Th17 populations. The two populations differentiate from a common progenitor CD4+ T cell population. IL-6, in concert with TGF-β, drives the differentiation of Th17 cells, while IL-6 blocks differentiation of Tregs [30,46,47]. IL-2, a product of activated T helper cells, inhibits the differentiation of CD4+ progenitor cells into mature Th17 cells while expanding the population of Tregs [48]. IL-2 is constitutively expressed at low levels by differentiating, but not mature Th17 cells [49]. Thus, the increase in IL-6 in placentas from infected viable fetuses (Figure 2C), along with the stable TGF-β (Figure 2F) and IL-2 (Figure 2B) expression in this group, had the potential to create a Th17 dominant environment. We did not find evidence of such a result. While the expression patterns of Treg and Th17 cytokines were not clearly predictive of a functional outcome in the placentas, the hallmark cytokines TGF-β and IL-17a, respectively, were discordantly coexpressed in this same group. When fetuses were nonviable, placental coexpression of IL-2 and IL-17a was aberrant. When infected groups included nonviable offspring (infected nonviable and all infected), IL-1β expression was discordant with all other cytokines. This result is most likely because expression of all other cytokines was significantly affected by fetal nonviability while IL-1β expression remained stable, producing a cytokine imbalance. These results indicate that infection may have perturbed Treg and/or Th17 populations, consistent with the reduction in expression of their respective intranuclear markers, FoxP3 and RORγ. A plausible explanation for this occurrence is that even in an environment that would favor Th17 cells, FIV infection reduces the two populations either by direct replication in the cells [31,32] or in a manner similar to the reduction that occurs with HIV or SIV disease progression [33,34].

Another putative placental cell population that may play a role in altered placental immunity in the FIV-infected cat model is the uterine NK (uNK) cell. The greatest concentration of leukocytes in the placenta (30-40% of all stromal cells) is present during early pregnancy, and 70% of those leukocytes are uNK cells. These cells play an important role in proper spiral artery remodeling and extravillous trophoblast invasion (reviewed in [50]). Much of the placental RNA isolated in this study may be derived from this cell population. Our data indicate significant changes in expression of IL-1β, IL-10, and TGF-β, also uNK products. Exploration of the role of uNK cells in placental immunopathology in the FIV-infected cat model was beyond the scope of the present study, but it is an obvious direction for future research.

FIV gag RNA was detected in representative placental tissues from queens included in this study [21]. FIV gag and cytokine gene expression neither positively nor negatively correlated in infected viable and infected nonviable groupings, suggesting that cytokine expression levels may not have impacted virus replication or vice versa. However, we saw a trend toward a negative correlation that did not reach significance in the infected nonviable group (n = 6; data not shown); yet, when all infected specimens were combined (n = 13), FIV gag versus IL-6, IL-10, and IL-17a were negatively correlated. Most likely the increased statistical power gained from the inclusion of more samples in the analysis revealed a negative impact of viral infection on cytokine gene expression. The mechanism of infection-induced pregnancy failure is unresolved, but placental immunopathology in the infected animal is a likely contributor.

## Conclusions

Reduced FoxP3 and RORγ and aberrant cytokine expression provide evidence of Treg and Th17 cell dysfunction in the placentas of FIV-infected cats, particularly those associated with fetal nonviability. Isolation of these cells to obtain pure populations for gene expression analyses is essential to determine with precision the direct impact of infection on the cell dynamics. We attempted to microdissect these cells from frozen placental specimens to extract their RNA for gene expression analyses, but we were unsuccessful in obtaining sufficient intact RNA to achieve this goal. Given the current understanding of the importance of these cell populations on pregnancy outcome, the vulnerability of these cells to lentivirus infection, and the negative impact of lentiviral infection on fetal viability, additional study of the impact of maternal lentivirus infections on placental Treg and Th17 cell dynamics is warranted.

## Methods

### Animals and virus

Female cats (*Felis domesticus*) were reproductively mature and specific pathogen-free (SPF). The animals were obtained from a commercial cattery. Ten cats were inoculated intravenously with feline plasma (1 mL) containing FIV-B-2542 at approximately 1 × 10^4^ virions per mL; ten uninoculated cats were used as controls [7]. Whole blood needed for plasma and peripheral blood leukocytes was collected into Vacutainer® tubes containing heparin; whole blood needed for serum was collected into Vacutainer® tubes without anti-coagulant. Animals were bled at weekly to monthly intervals until delivery of kittens. FIV infection was confirmed by performing standard PCR targeting the FIV gag gene and serology to detect FIV specific antibodies [8]. Queens were allowed to breed naturally with SPF males. Pregnancies were confirmed by ultrasonography at week 3–4 gestation (early term), then fetuses were delivered by cesarean section. Inoculated animals had been infected from 9.5 to 13.5 months (mean 11.4 months) at the time of delivery. Fetal and placental tissues were collected from all animals, snap frozen in liquid nitrogen, and stored at −80°C. Infected animals were euthanized following delivery; control cats were spayed and released for adoption after recovery. All animal use was approved by the Institutional Animal Care and Use Committee of Mississippi State University. Animal tissues used in these studies are identified in Table 1.

### RNA extraction from whole placental tissue

All manipulations of frozen placental tissue were performed under RNAse free conditions. Total RNA was isolated from whole tissue samples by placing 50–100 mg of thawed placental tissue into Eppendorf tubes and adding 1.0 mL of Trizol Reagent (Invitrogen, Carlsbad, CA). Tissue was homogenized with a sterile pestle for 5 min. Two hundred μL of chloroform were added; the tubes were shaken and then vortexed vigorously for 15 sec, and tubes were allowed to incubate at room temperature for 2–3 min. The tubes were centrifuged for 15 min at 12,000 × g and 4°C in a MX-305 High Speed Refrigerated Micro Centrifuge (Tomy, Menlo Park, CA). The aqueous phase was transferred to a new tube, and 0.5 mL of isopropanol was added. The tubes were incubated at room temperature for 10 min and then centrifuged for 10 min at 12,000 × g and 4°C. The RNA pellet was washed twice with 1.0 mL of 75% ethanol followed by centrifugation at 7,500 × g. The supernatant was removed and the RNA pellet was air dried for 5–10 minutes and then redissolved in DEPC-treated water. Dissolved RNA concentration was determined using a NanoDrop 1000 (Thermo Scientific, Waltham, MA) and stored at −20°C.

### cDNA conversion

Isolated RNA was converted to cDNA using a High Capacity Reverse Transcription cDNA conversion kit (Applied Biosystems, Carlsbad, CA). RNA in the amount of 1.0-2.0 μg was diluted in 10 μL of DEPC-treated water and added to a reverse transcription mixture containing: 2 μL 2X RT buffer, 0.8 μL of 25X dNTP mix, 1.0 μL of reverse transcriptase, 2.0 μL of random primers, 1.0 μL RNase inhibitor, and 3.2 μL of DEPC-treated water. Reverse transcription was carried out in reaction volumes of 20 μL using the following protocol: 25°C for 10 min, 37°C for 120 min, 85°C for 5 sec.

### Quantification of immunomodulator expression

cDNA was used in quantitative PCR (qPCR) to quantify expression of relevant pro- and anti-inflammatory cytokines IL-1β, IL-2, IL-6, IL-10, IL-17a, and TGF-β, Th17 cell marker RORγT, Treg cell marker FoxP3, FIV gag, and β-actin. One μL of 20X Taqman Gene Expression Assay, 9 μL (500 ng) of cDNA template (in Rnase-free water), and 10 μL of 2X Taqman gene expression master mix were added to 96-well optical plates (Applied Biosystems). All PCR reactions were performed in triplicate. Individual primers and probes present in the Taqman Gene Expression Assays are presented in Table 2. A 2-stage qPCR program was used: 50°C for 2 min and 95°C for 10 min, followed by 95°C for 15 sec and 60°C for 1 min (40 cycles). Individual immunomodulator and viral marker Ct values were normalized to β-actin, and the normalized Ct values were subtracted from a negative end point to invert the data for ease of interpretation. FIV gag gene expression was previously reported [21].

**Table 2:** Primer/probe sequences used in gene expression assays

**Gene**	**Primer/Probe**	**Sequence (5′-3′)**
β-actin	Probe	/56-FAM/ATG GAG AAG /ZEN/ATCTGGCACCACACCTT/31ABkFQ/
	Forward	ATCGAACACGGC ATT GTCACCAAC
	Reverse	AGT CAT CTT CTC ACGGTTGGCCTT
IL-1β	Probe	/56′FAM/AAA GAG CCT /ZEN/GGTGCTGTCTGG CTC AGA/31ABkFQ/
	Forward	TGATGCAGC CAT ACAGTCACAGGA
	Reverse	CAAAGCTCATGCGGAACACCACTT
IL-2	Probe	/56-FAM/ACT CGTCAC /ZEN/AAA CAG TGCACCTGCTT/31ABkFQ/
	Forward	TAACCTCAA CTC CTGCCACCACAA
	Reverse	TTG CTC CAG CTGTTGCTGTGTTTC
IL-6	Probe	/56-FAM/AACCGT AGA /ZEN/AGTTGGCCTGCAGCT AA/31ABkFQ/
	Forward	TCC AGA TGCTGAAGCGTAAGGGAA
	Reverse	AAGGGT GAG GTG ATT GTTGTGTGC
IL-10	Probe	/56-FAM/ACCAGGTCC /ZEN/TTGCTG GAG GACTTT AA/31ABkFQ/
	Forward	TTTCAAACCAAGGAC GAG CTGCAC
	Reverse	AACTGGATCATCTCGGACAAGGCT
IL-17a	Probe	/56-FAM/GTGAGGACA /ZEN/GGA ATA GCATTCCCA CA/31ABkFQ/
	Forward	TCC CAT TCAGTT CCC ATC ACT GCT
	Reverse	TCGGTAGTTGGG CAT CCTGGATTT
TGF-β	Probe	/56-FAM/AGCAATAAT /ZEN/TCCTGGCGCTAC CTC AGC A/31ABkFQ/
	Forward	AGCACGTGGAGCTGTACC AGA AAT
	Reverse	TCCAGTGACATC AAA GGA CAG CCA
Gag	Probe	/56-FAM/AAGAACAAC /ZEN/AAG CAG AAGCAA GAT TTGCAC/31ABkFQ/
	Forward	CAG GGTGCGCTG CAG ATA AAG AAA
	Reverse	ATG CTC TAC ACT GCATCC TAG CTG
FoxP3	Probe	/56-FAM/AGC CTA CAC /ZEN/AAA TGCTTTGTGCGG GT/31ABkFQ/
	Forward	GGTTCACACGCATGTTTGCCTTCT
	Reverse	ACT CGA ATT CAT CCACGGTCCACA
RORγT	Probe	/56-FAM/AGA AATGCC /ZEN/TTGCCG TAG GGATGT CT/31ABkFQ/
	Forward	AACCGTTGCCAACACTGTCGATTG
	Reverse	ACGCCATGCCACTCTACA ACT CAA

FAM = 6-carboxyfluorescein; fluorophore; ZEN = 5′ quencher; ABkFQ = Iowa Black FQ; 3′ quencher.

### Statistical analysis

Both Wilcoxon signed rank test (http://www.wessa.net) and single factor ANOVA were used to compare gene expression levels in all sample groups. Spearman rank correlation was performed to assess the pairwise relationship of cytokines and viral RNA expression (http://www.wessa.net). In all analyses, p values ≤ 0.05 were considered significant.

## Competing interests

The authors declare that they have no competing interests.

## Authors’ contributions

LBC performed all experiments, collected and analyzed data, assisted with data interpretation, and drafted the manuscript. CEB assisted with experimental design and the performance of qPCR, aided in the interpretation of data, and critically evaluated the manuscript. KSC conceived of the study, secured funding, assisted with analysis and interpretation of data, and finalized the manuscript. All authors read and approved the final manuscript.
